# A New Pyrroloquinoline-Derivative-Based Fluorescent Probe for the Selective Detection and Cell Imaging of Lysine

**DOI:** 10.3390/ph15040474

**Published:** 2022-04-13

**Authors:** Bing Yang, Jiahua Zhou, Xu Huang, Zhongping Chen, Shu Tian, Yujun Shi

**Affiliations:** 1 School of Chemistry and Chemical Engineering, Nantong University, Nantong 226019, China; 1908320018@stmail.ntu.edu.cn (J.Z.); syj@ntu.edu.cn (Y.S.); 2 Institute of Special Environmental Medicine, Nantong University, Nantong 226019, China; 2023310004@stmail.ntu.edu.cn; 3 School of Textile and Clothing, Nantong University, Nantong 226019, China

**Keywords:** lysine detection, fluorescent probe, biological imaging, pyrroloquinoline structure, natural mineral water for drinking

## Abstract

In this paper, a new pyrroloquinoline-derivative-based fluorescent probe, **PQP-1**, was prepared for the selective detection of Lys in living cells and natural mineral water for drinking. **PQP-1** exhibited high selectivity, low limit of detection, and a wide pH range. **PQP-1** could be successfully applied for imaging Lys in living cells and in natural mineral water for drinking. We expect that **PQP-1** will expand the detection reaction mechanism and the practical biological applications of Lys.

## 1. Introduction

l-lysine is an essential amino acid for mammals and human beings which cannot be synthesized by the body itself and can only be obtained from food [1]. However, lysine is very low in cereals, so it is also known as the first limiting amino acid. l-lysine plays an important role in the regulation of protein synthesis [2,3] and energy metabolism [4,5], and it can improve mineral absorption [6] and bone growth [7], enhance immunity [8], and relieve anxiety [9]. The WHO/FAO/UNU Expert Committee had established a l-lysine requirement of 30 mg·kg^−1^·d^−1^ [10], which is now widely accepted. Thus, the development of analytical methods for the detection of lysine is of significance for medical and biological research.

Some analytical techniques have been used to detect lysine, including colorimetric method [11,12,13,14], liquid chromatography [15] and thin-layer chromatography [16], mass spectrometry [17], luminescent sensors [18], amperometric biosensors [19,20], electrochemical detection [21,22,23,24], and fluorescent probes [25,26,27,28,29,30,31,32,33,34,35,36,37,38,39,40,41,42,43]. In these methods, fluorescent probes feature high sensitivity and selectivity—satisfactory capability compared with other complicated methods—having been studied by more researchers. However, many reported fluorescent probes simultaneously detect multiple amino acids; which is to say that the selectivity of the fluorescent probe to lysine requires further research. Additionally, organic micromolecule fluorescent probes for detection of lysine have been scarcely reported.

In this study, a new pyrroloquinoline-derivative-based fluorescent probe, **PQP-1**, was successfully synthesized. The probe **PQP-1** can specifically recognize Lys and not react equally with homocysteine (Hcy), glutathione (GSH), glucose (GLu), various ions, and other amino acids. Afterwards **PQP-1** was used to monitor Lys in HeLa cells and in Nongfu natural mineral water for drinking. Comparing with other reported probes listed in Table S1, **PQP-1** features relatively simple structure, and can be easy to synthesize. In addition, the detection of **PQP-1** to Lys can be performed in water without a large amount of additional organic solvents. Most of all, **PQP-1** exhibited a high selectivity toward Lys, low limit of detection, and wide pH range. At the same time, **PQP-1** can be successfully applied for cell imaging and real water samples, but not all reported probes can be used.

## 2. Results and Discussion

### 2.1. Synthesis of the Probe ***PQP-1***

**Figure 1:**
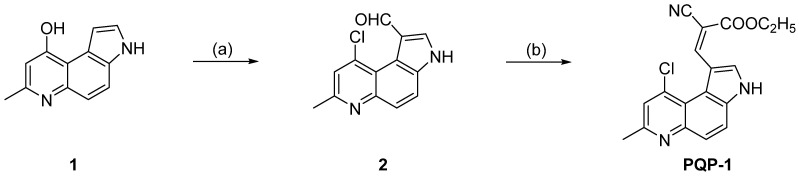
Synthetic route of **PQP-1**. Reagent and condition: (**a**) POCl_3_, DMF, 30 °C, 8 h, 90%; (**b**) ethyl cyanoacetate, EtOH, reflux, 5 h, 85%.

The probe **PQP-1** was prepared from compound **1** according to the route in Figure 1. Its structure was confirmed (^1^H NMR and ^13^C NMR seen in Figures S1–S3).

### 2.2. Fluorescent Response of Probe ***PQP-1*** to Lysine

**Figure 2:**
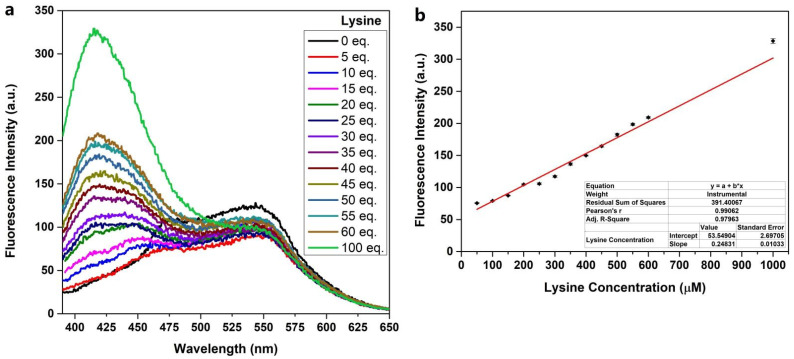
(**a**) The fluorescence spectra of **PQP-1** (10 μM) in deionized water after treatment with L-lysine (0–1000 μM) for 30 min; (**b**) The fluorescence intensity at around 420 nm has a good linear relationship with L-lysine concentrations (50–1000 μM). The data come from three parallel experiments.

The fluorescence quantum yield (Φ_u_) of probe **PQP-1** is 0.05. Based primarily on optimization, 10 μM was selected as the testing concentration of **PQP-1** and 30 min was the testing reaction time. With measuring conditions in hand, the varying regularity of the fluorescence spectroscopy have been estimated in the absence and presence of l-lysine in deionized water. Under the excitation wavelength of 335 nm, the fluorescence spectra of **PQP-1** detecting l-lysine suggested the strong emission peak at around 420 nm appeared after the addition of l-lysine (Figure 2). The fluorescence enhancement also exhibited a linearly increasing relationship to the concentration of l-lysine (50–1000 μM). According to the equation the detection limit (LOD) = 3σ/k, the detection limit was calculated to be 21.89 nM.

Then, the pH-dependent fluorescent response experiments of **PQP-1** to lysine were carried out. As we can see from Figure S4, the fluorescence intensity of **PQP-1** remained stable in the 5.0–11.0 pH range. After 600 μM of l-lysine was added, the response of **PQP-1** can hold steady at pH 6.0–9.0.

### 2.3. Selective Detection for Lysine

**Figure 3:**
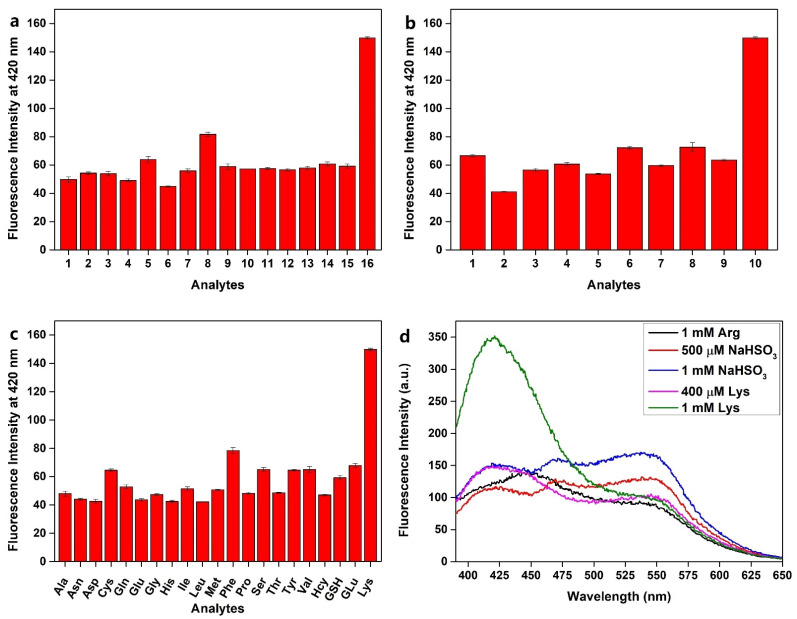
The selectivity of **PQP-1** for Lys compared with various ions, Hcy, GSH, glucose (GLu), and other amino acids. (**a**) **PQP-1**: 10 μM, Lys: 400 μM, other: 1 mM, (1) F^−^, (2) Cl^−^, (3) Br^−^, (4) I^−^, (5) NO_3_^−^, (6) NO_2_^−^, (7) HCO_3_^−^, (8) CO_3_^2−^, (9) SO_4_^2−^, (10) S_2_O_3_^2−^, (11) S^2−^, (12) Ac^−^, (13) ^−^OOCCOO^−^, (14) EDTA^2−^, (15) H_2_O_2_, (16) Lys; (**b**) **PQP-1**: 10 μM, Lys: 400 μM, other: 1 mM, (1) K^+^, (2) Na^+^, (3) Ca^+^, (4) Ba^2+^, (5) Cu^2+^, (6) Mn^2+^, (7) Zn^2+^, (8) Mg^2+^, (9) NH_4_^+^, (10) Lys; (**c**) **PQP-1**: 10 μM, Lys: 400 μM, other: 1 mM; (**d**) **PQP-1**: 10 μM.

The selectivity was discussed through the comparison of the fluorescence intensity in the presence of various anions, metal cations, amino acids, GSH, Hcy, and GLu. As shown in Figure 3a–c, except for lysine, none of these competitive species led to obvious fluorescence response. However, cyano-based probes were usually applied in the detection of sulfur dioxide derivatives (HSO_3_^−^/SO_3_^2−^), and reports suggested the amino acid Arg had similar response to Lys, so HSO_3_^−^ and Arg were investigated for evaluating the selectivity of **PQP-1** to Lys. The results in Figure 3d demonstrated the response peak of 1 mM Arg appeared at 450 nm rather than 420 nm (the response peak of Lys); meanwhile, three peaks at 420, 475, and 550 nm were observed in the fluorescence spectra after the addition of 500 μM or 1 mM NaHSO_3_. In addition, the peak at 420 nm after the addition of 1 mM NaHSO_3_ was almost as high as the peak after the addition of 400 μM l-lysine but far below the peak after the addition of the same concentration of l-lysine. Therefore, we can conclude that HSO_3_^−^ and Arg cannot react with **PQP-1** as well as Lys. These results showed that **PQP-1** exhibited high selectivity.

### 2.4. Proposed Response Mechanism

**Figure 4:**
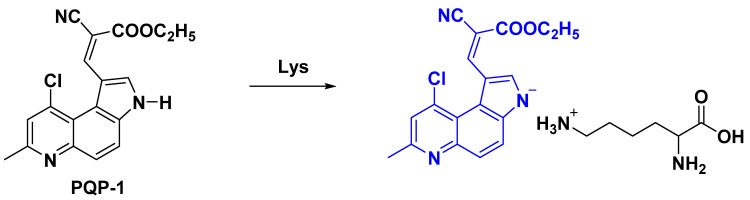
Proposed response mechanism between **PQP-1** and lysine.

The response mechanism between **PQP-1** and lysine was shown in Figure 4. *ε*-Amino group in lysine structure can capture the proton bonded to nitrogen of the pyrrole structure on **PQP-1**, increase the electron cloud density of the **PQP-1** structure, and change the electronic configuration, which can bring about the new fluorescence response signal.

**Figure 5:**
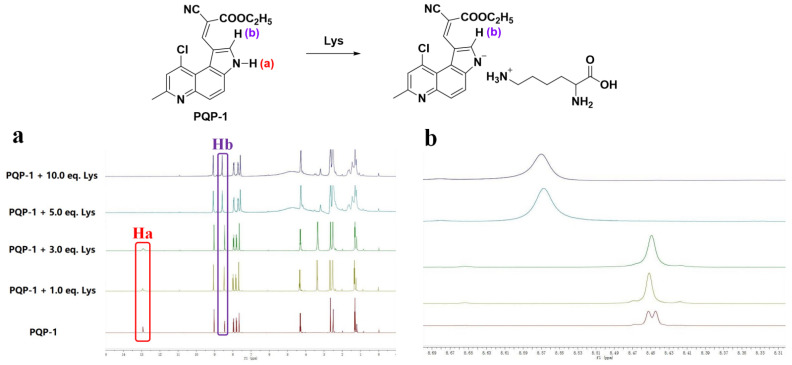
^1^H NMR titration experiments of **PQP-1** in DMSO-*d*_6_ (0.55 mL) on lysine. **PQP-1**: 0.0051 g, 15 μmol. (**a**) The comparison of ^1^H NMR spectra of **PQP-1** and the mixture after adding different concentrations of lysine. (**b**) The changes of peak type of **Hb**.

The response mechanism as mentioned in Figure 4 was confirmed by ^1^H NMR titration results in Figure 5. There are at least two pieces of evidence. On one hand, with increase in the concentration of Lys, the peak of the hydrogen **Ha** on the pyrrole nitrogen decreased gradually and disappeared at last (Figure 5a). On the other hand, the peak type of hydrogen **Hb** in aromatic ring adjacent to nitrogen in the pyrrole structure changed from doublet into singlet. These data suggested that the proton **Ha** had been abstracted in **PQP-1** during the detection of lysine, which could support the proposed response mechanism.

### 2.5. Imaging Study

**Figure 6:**
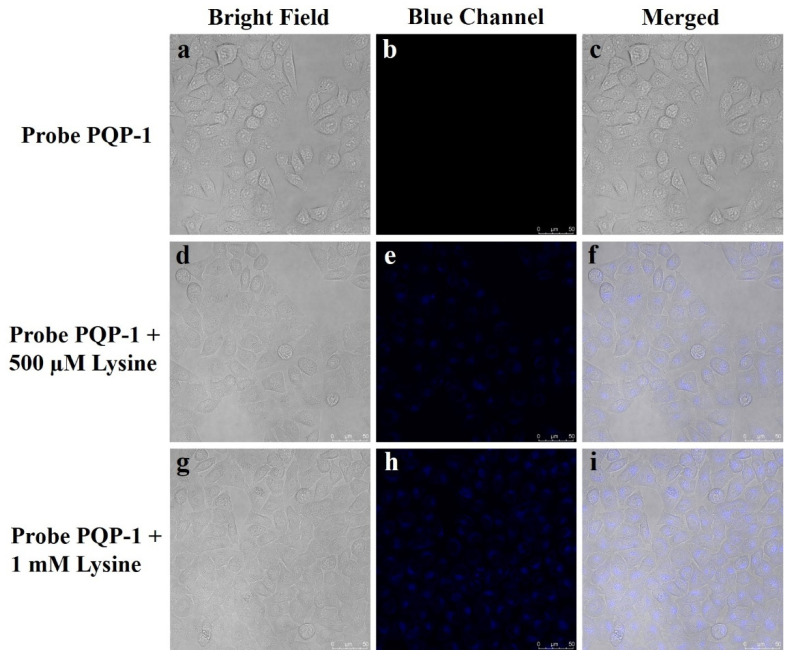
Fluorescence imaging of lysine in living HeLa cells. (**a**–**c**) images of HeLa cells treated with **PQP-1** (10 μM) for 30 min; (**d**–**f**) images of HeLa cells pretreated with **PQP-1** (10 μM) for 30 min, then incubated with 500 μM L-lysine for an additional 30 min; (**g**–**i**) images of HeLa cells pre-incubated with **PQP-1** (10 μM) for 30 min, then treated with 1 mM l-lysine for an additional 30 min. Excitation at 405 nm; Scale bar: 50 μm.

The intracellular performance in monitoring l-lysine was further revealed on a confocal fluorescent microscope (Figure 6). After HeLa cells were incubated with **PQP-1** (10 μM) for 30 min, there was no obvious fluorescent signal (Figure 6a–c). When the cells were incubated with 10 μM of the probe **PQP-1** for 30 min and subsequently incubated with 500 μM of l-lysine (Figure 6d–f) and 1 mM (Figure 6g–i), respectively, the enhancement of the fluorescence signal was observed compared with that of the control. Notably, the fluorescence signal increased in a dose-dependent manner. In a word, these observations indicate that **PQP-1** can detect lysine in living cells.

### 2.6. Detection of Lysine Concentrations in Natural Mineral Water for Drinking

Not only can lysine be mixed with various vitamins to compound nutritional supplements, but it can improve the performance of some drugs to enhance the efficacy of drugs. These nutritional supplements and drugs are commonly used in tablet form. On the other hand, natural mineral water for drinking is daily water, which is convenient for sampling. In order to study the effect of mineral water for drinking on these tablets, **PQP-1** was further applied to detect l-lysine in natural mineral water for drinking. As shown in Table 1, testing results of **PQP-1** to l-lysine was found to be consistent with the real adding amount of l-lysine under the standard testing conditions. The range of recovery was between 96.65% and 101.93%, indicating that the natural mineral water for drinking did not influence the recognition of **PQP-1** toward Lys.

## 3. Materials and Methods

### 3.1. Materials and Apparatus

5-Aminoindole and ethyl cyanoacetate were purchased from Bide Pharmatech Ltd., Shanghai, China. Ethyl acetoacetate was purchased from Shanghai Macklin Biochemical Co., Ltd., Shanghai, China. Phosphorus oxychloride (POCl_3_) was purchased from Shanghai xianding Biotechnology, Shanghai, China. Dimethyl formamide (DMF) was purchased from Sinopharm Chemical Reagent Co., Ltd., Shanghai, China. The purchased chemicals were directly used. The purification of products was performed with silica gel column chromatography (silica gel: 200–300 mesh, Qingdao Ocean Chemical Co. Ltd., Qingdao, China). HeLa cells (CCL-2, PRID: CVCL_0030) was obtained from ATCC.

Melting points were determined on a micro melting point apparatus (SGW X-4B, Shanghai, China) and uncorrected. ^1^H and ^13^C NMR spectra were measured with a Bruker AVANCE III HD 400M spectrometer (Zurich, Switzerland). Chemical shifts (*δ*) were shown in ppm (parts per million) with respect to TMS. Coupling constants (*J*) were reported in Hz. HRMS (High Resolution Mass Spectrometry) data were obtained from an AB Sciex TripleTOF 4600 System mass spectrometer (Framingham, MA, USA) with an ESI (electrospray ionization) source.

The UV–vis absorption measurement was conducted on a Shimadzu UV-3600 spectrometer (Tokyo, Japan). All fluorescence tests were obtained from a Hitachi F-7000 Fluorescence Spectrometer (Tokyo, Japan). The cell imaging experiments were accomplished on a Leica TCS SP8 STED 3X confocal fluorescent microscope (Wetzlar, Germany).

### 3.2. Preparation of the Probe ***PQP-1***

The preparation process of **PQP-1** was shown in Figure 1.

According to previous reports [44,45], 3*H*-9-Hydroxy-pyrrolo[3,2-*f*]quinoline (**1**) and 3*H*-9-chloro-7-methyl-1-formyl-pyrrolo[3,2-*f*]quinoline (**2**) were synthesized. Compound **1** can develop from condensation and cyclization of 5-aminoindole with ethyl acetoacetate, and be used after filtration without purification. Then, compound **2** was prepared from the Vilsmeier–Haack formylation reaction of compound **1** with POCl_3_ and DMF. The pure compound **2** can be obtained by silica gel column.

To an ethanol solution (25.0 mL) of aldehyde compound **2** (0.2445 g, 1.0 mmol) was added ethyl cyanoacetate (0.17 mL), and the reaction liquid was heated to reflux for 5 h while stirring. After the consumption of the reaction was confirmed, the reaction mixture was evaporated under reduced pressure. The crude product was purified by silica gel chromatography to give a yellow solid (**PQP-1**, 0.2887 g, yield: 85%). m.p. 203.0–204.0 °C. ^1^H NMR (400 MHz, DMSO-*d*_6_) *δ* 1.30 (t, 3H, *J* = 7.2 Hz), 2.64 (d, 3H, *J* = 1.2 Hz), 4.30 (q, 2H, *J* = 7.2 Hz), 7.62–7.65 (m, 1H), 7.78–7.82 (m, 1H), 7.93–7.97 (m, 1H), 8.45 (d, 1H, J = 2.4 Hz), 9.02–9.04 (m, 1H), 12.90 (s, 1H). ^13^C NMR (100 MHz, DMSO-*d*_6_) δ 14.2, 23.8, 61.7, 93.9, 112.3, 117.1, 117.5, 118.2, 119.6, 122.6, 125.8, 130.8 (d, *J* = 15.0 Hz), 135.3, 137.5, 147.4, 153.5, 155.9, 162.9. HRMS (ESI-TOF) *m/z*: [M+H]^+^ Calcd. for C_18_H_15_ClN_3_O_2_ 340.0847, Found 340.0843.

### 3.3. Testing Conditions

The solution of probe **PQP-1** in DMSO and deionized water (V:V = 1:4) was diluted for testing. The deionized water was used to prepare the testing solution of other analytes. λ_ex_ = 335 nm, slit: 5 × 5 nm.

### 3.4. Calculation of the Fluorescence Quantum Yield

The sulfuric acid solution (0.1 M) of quinine sulfate (1 µM, Φ = 0.54, λ_ex_ = 360 nm) used as the standard, the following equation was used to calculate the fluorescence quantum yield (FQY) Φ_u_:

Φ_u_ = [(A_s_F_u_n^2^)/(A_u_F_s_n_0_^2^)]Φ_s_.

Φ_s_ is the quantum yield of quinine sulfate; A_s_ and A_u_ must be lower than 0.05, refer to the absorbance of the standard and **PQP-1** (1 µM) at the respective excitation wavelength; F_s_ and F_u_ represent the integrated emission band areas; n and n_0_ are the refractive indexes of water and sulfuric acid solution (0.1 M), respectively.

Φ_s_ = 0.54, A_s_ = 0.009, F_s_ = 119.159, n_0_ = 1.3330;
A_u_ = 0.0207, F_u_ = 27.036, n = 1.3330;
Quantum yield: Φ_u_ = 0.05.

### 3.5. Calculation of the Detection Limit

The following equation was used to calculate the detection limit (LOD):

LOD = 3σ/k

where σ is the standard derivation of 25 blank **PQP-1** solutions, k refers to the slope between the fluorescence intensity at around 420 nm and a series of concentrations of L-lysine.

### 3.6. Imaging Study

HeLa cells were cultured for 12 h in a humidified atmosphere carrying 5% CO_2_. The cells were washed by PBS three times, then used for cell imaging.

### 3.7. Water Sample Preparation

The natural mineral water for drinking was derived from Nongfu barreled natural mineral water for drinking. The natural mineral water was directly used as the solution system in the tests instead of above deionized water.

## 4. Conclusions

In general, we prepared a new fluorescent probe, **PQP-1**, containing a pyrroloquinoline structure for the selective detection of Lys. Research results suggested that **PQP-1** had a high selectivity to Lys, low limit of detection, and wide pH range. Moreover, **PQP-1** could be successfully applied for the living cell imaging of Lys. Finally, **PQP-1** has been used in natural mineral water for drinking. Furthermore, we expect that **PQP-1** will broaden the reaction mechanism of Lys detection as well as its biological applications.

## Figures and Tables

**Table 1:** Detection of l-lysine concentrations in natural mineral water for drinking. l-lysine with known concentrations was added into the natural mineral water for drinking. The concentration of **PQP-1** was 10 μM. The data come from three parallel experiments.

Entry	Added Concentrations (μM)	Detected Concentrations (μM)	Recovery (%)
1	80	80.37 ± 1.43	100.46
2	220	224.25 ± 2.86	101.93
3	490	484.95 ± 0.45	98.97
4	750	724.84 ± 3.04	96.65

## Data Availability

Data is contained within the article or supplementary material.

## Supplementary Materials

The following supporting information can be downloaded at: https://www.mdpi.com/article/10.3390/ph15040474/s1, Figure S1: ^1^H NMR spectra of **PQP-1**; Figure S2: ^13^C NMR spectra of **PQP-1**; Figure S3: HRMS spectra of compound **PQP-1**; Figure S4: The variation of the fluorescence intensity at 420 nm of **PQP-1** (10 μM) at different pH values (from 5.0 to 11.0) in the absence (black) and presence (red) of L-lysine (600 μM); Table S1: The comparison of **PQP-1** and reported small-molecule fluorescent probes for Lys.
