# Supplementary material for: A New Pyrroloquinoline-Derivative-Based Fluorescent Probe for the Selective Detection and Cell Imaging of Lysine
Source: Pharmaceuticals (Basel). 2022 Apr 13;15(4):474. doi: 10.3390/ph15040474 (PMC9029482; doi:10.3390/ph15040474)
Supplement: Supplementary file 1 [file pharmaceuticals-15-00474-s001.zip › pharmaceuticals-1610598-supplementary.pdf]

Supporting Information

A New Pyrroloquinoline-Derivative-Based Fluorescent Probe for the Selective Detection and Cell Imaging of Lysine

Bing Yang [1,*], Jiahua Zhou [1], Xu Huang [2], Zhongping Chen [2,*], Shu Tian [1,*], and Yujun Shi [1,3]

[1] School of Chemistry and Chemical Engineering, Nantong University, Nantong 226019, China;
[2] Institute of Special Environmental Medicine, Nantong University, Nantong 226019, China;
[3] School of Textile and Clothing, Nantong University, Nantong 226019, China.
* Correspondence: yangbing111@ntu.edu.cn (B.Y.); chenzp@ntu.edu.cn (Z.C.); tian0429@ntu.edu.cn (S.T.)

1. NMR and HRMS spectra of PQP-1

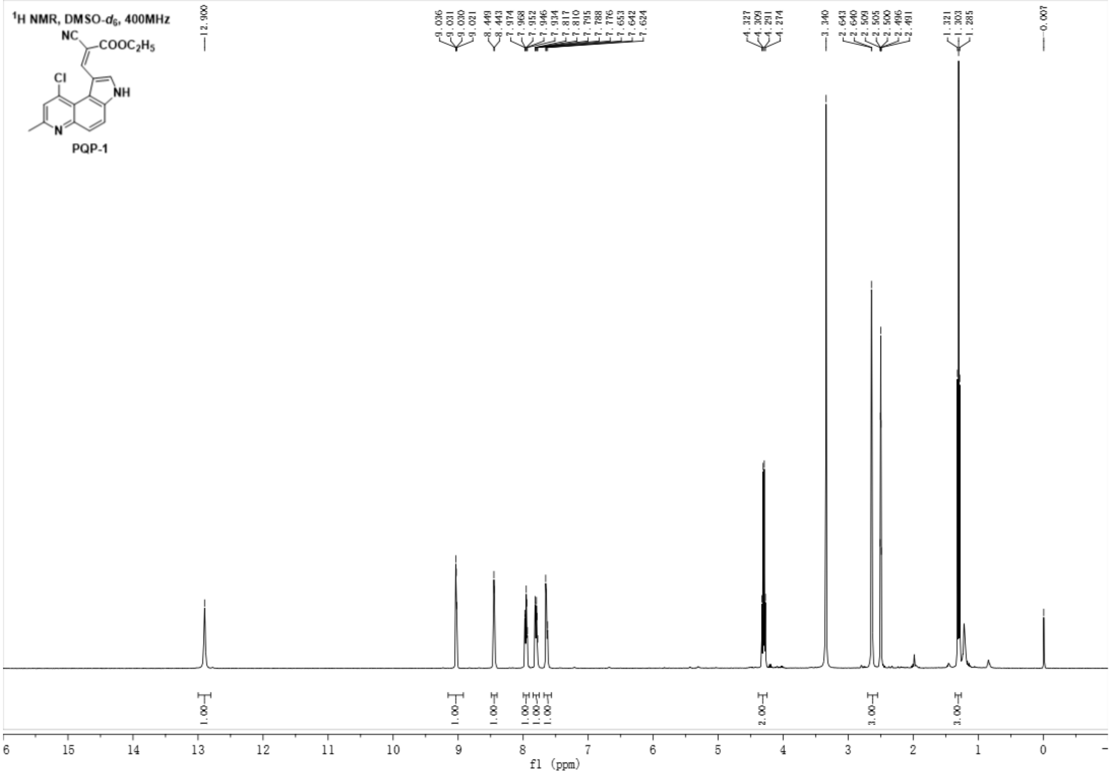

Figure S1. ^{1}H NMR spectra of **PQP-1**.

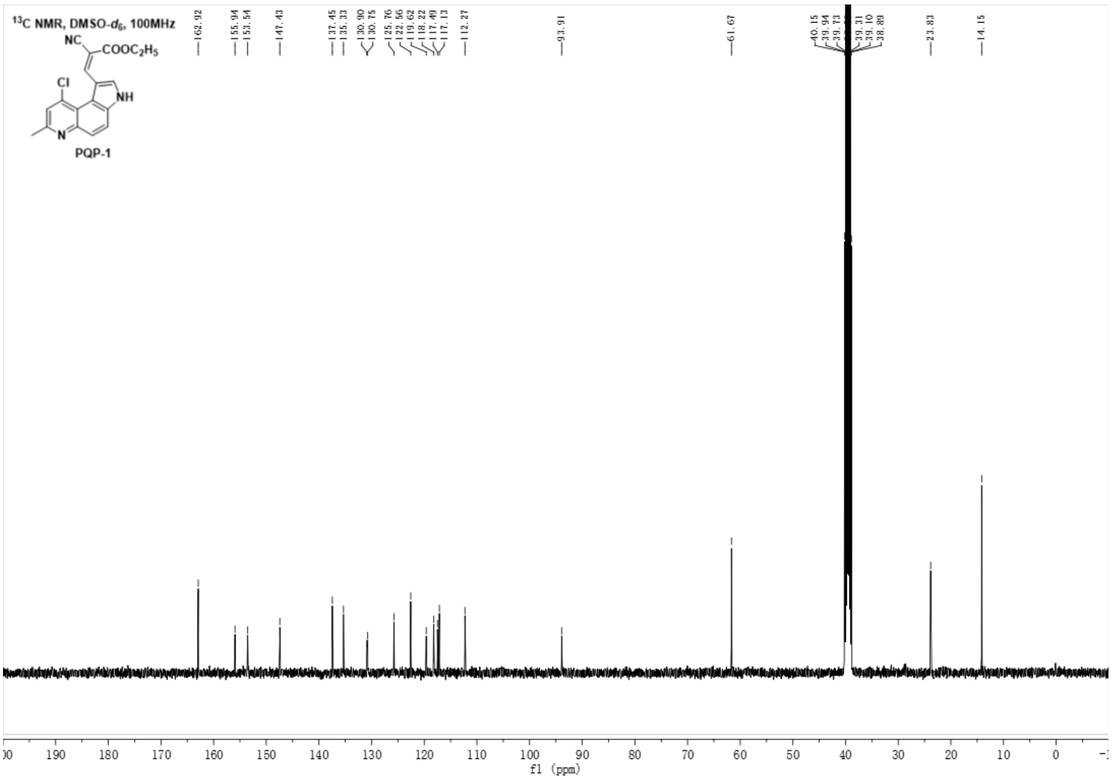

Figure S2. ^{13}C NMR spectra of **PQP-1**.

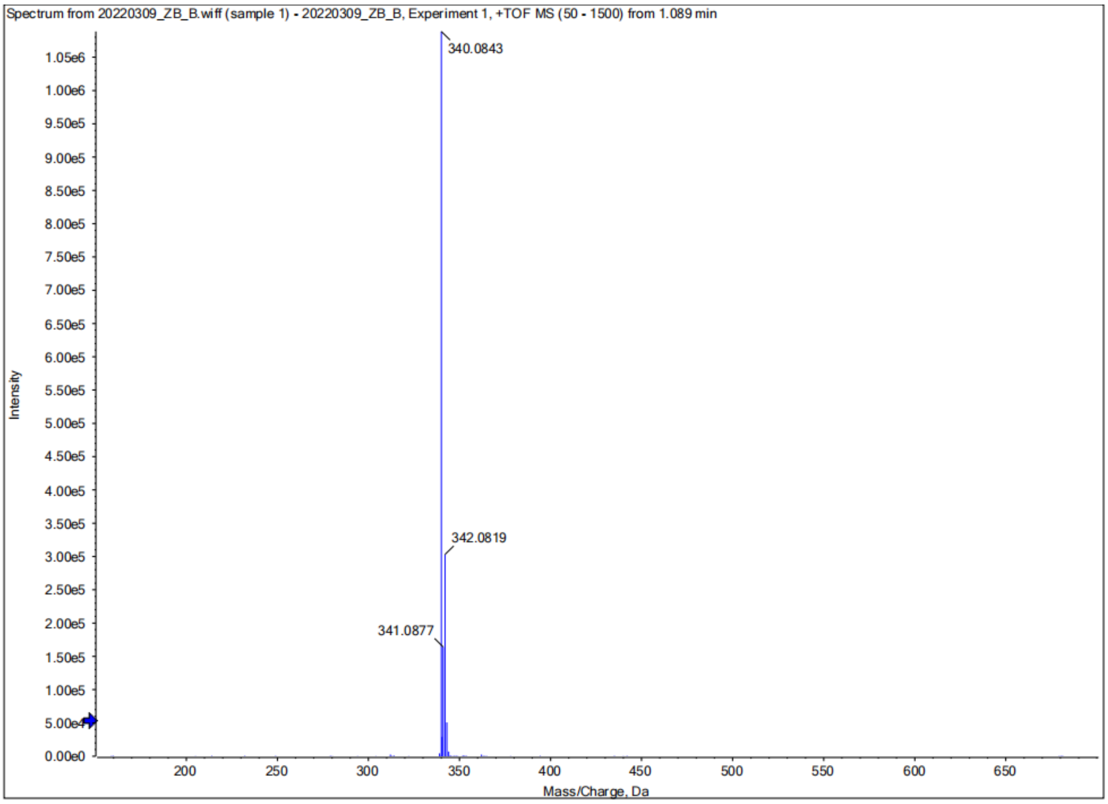

Figure S3. HRMS spectra of compound **PQP-1**.

2. Figures and tables

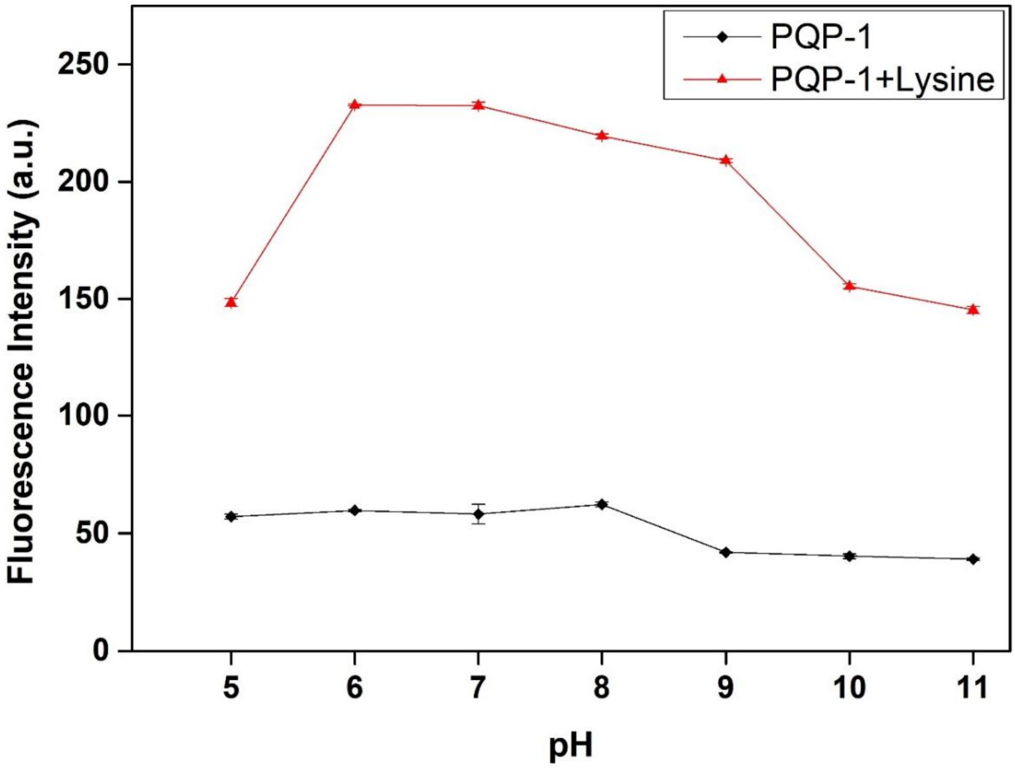

Figure S4. The varitation of the fluorescence intensity at 420 nm of **PQP-1** (10 μM) at different pH values (from 5.0 to 11.0) in the absence (black) and presence (red) of L-Lysine (600 μM).

Table S1. The comparison of **PQP-1** and reported small-molecule fluorescent probes for Lys.

No.	Structure	λ_{ex}/λ_{em} （nm）	Solvent	Detection concentration of probe (μM)	Detection time (min)	LOD （nM）	Selectivity	Application	Ref.
1	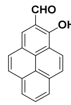	380/465	CH_3CN-HEPES buffer (0.01 M, pH 7.4) (1:9, v/v)	20	-	-	Lys	-	[1]
2	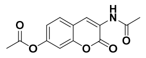	344/471	20 mM HEPEs, pH 7.4	5	30	-	Lys, Arg	-	[2]
3	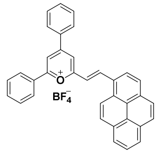	365/457	CH_3CN-H_2O (v:v, 1:1)	50	6	3610	Lys	Filter paper	[3]
4	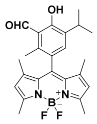	503/582	DMSO-HEPES buffer (1:9, v/v, 0.01 M, pH 7.4)	10	-	1	Lys	MDA-MB 231 cells	[4]
5	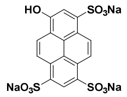	455/510	Water	5	-	3106	Lys, Arg	-	[5]
6	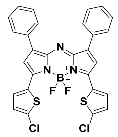	700/758	DMSO/H_2O (1/4, v/v, pH = 7.3)	1	10	-	Lys	PC3 cells	[6]

7	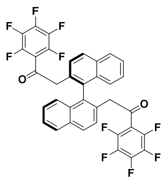	332/426	Water (1% DMF)	10	30	51000	Lys	-	[7]
8	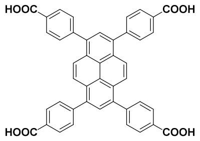	365/440	Water	0.02 mg/mL	Around 3 s	2200	Lys, Arg	-	[8]
9	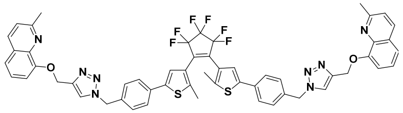	305/508	CH_3CN-H_2O (7/3, v:v)	20	-	19	Lys	Logic circuits	[9]
10	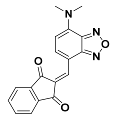	458/580	Water (30% DMF)	10	150	1100	Lys, Arg	-	[10]
11	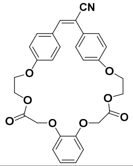	320/439	H_2O/DMSO (9:1)	1	-	134	Lys	HeLa cells; The urine for adults and children	[11]
12	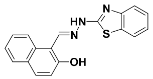	450/515	DMSO/H_2O (6:4, v/v)	10	< 2 s	36.9	Lys, Arg	-	[12]
13	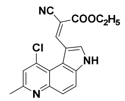	**335/420**	**Water**	**10**	**30**	**21.89**	**Lys**	**HeLa cells; Natural mineral water for drinking**	**This work**

References

1. Zhou, Y.; Won, J.; Lee, J.Y.; Yoon, J. Studies leading to the development of a highly selective colorimetric and fluorescent chemosensor for lysine. *Chem. Commun.* **2011**, *47*, 1997-1999.
2. Hou, J.-T.; Li, K.; Liu, B.-Y.; Liao, Y.-X.; Yu, X.-Q. The first ratiometric probe for lysine in water.*Tetrahedron* **2013**, *69*, 2118-2123.
3. Qian, X.; Gong, W.; Wang, F.; Lin, Y.; Ning, G. A pyrylium-based colorimetric and fluorimetric chemosensor for the selective detection of lysine in aqueous environment and real sample. *Tetrahedron Lett.* **2015**, *56*, 2764-2767.
4. Adhikari, S.; Ghosh, A.; Mandal, S.; Guria, S.; Banerjee, P.P.; Chatterjee, A.; Das, D. Colorimetric and fluorescence probe for the detection of nano-molar lysine in aqueous medium. *Org. Biomol. Chem.* **2016**, *14*, 10688-10694.
5. Bhosale, R. S.; Shitre, G. V.; Kumar, R.; Biradar, D. O.; Bhosale, S. V.; Narayan, R.; Bhosale, S. V. A 8-hydroxypyrene-1,3,6-trisulfonic acid trisodium salt (HPTS) based colorimetric and green turn-on fluorescent sensor for the detection of arginine and lysine in aqueous solution. *Sens. Actuators, B* **2017**, *241*, 1270-1275.
6. Jiang, X.D.; Yue, S.; Jia, L.; Li, S.; Li, C.; Li, Q.; Xiao, L. NIR fluorescent azaBODPIY-based probe for the specific detection of L-lysine. *Chemistryselect* **2018**, *3*, 7581-7585.
7. Zhao, H.; Li, Y.; Cao, Y.; Gong, G.; Zhou, Y.; Gao, X. X.; Pu, L.; Zhao, G. Spectroscopic studies of a BINAM-based sensor: Highly selective fluorescent recognition of lysine in water solution through a nucleophilic substitution reaction. *Tetrahedron Lett.* **2019**, *60*, 1238-1242.
8. Hao, J.; Wang, M.; Wang, S.; Huang, Y.; Cao, D. Dissolution-enhanced emission of 1,3,6,8-Tetrakis(*p*-benzoic acid)pyrene for detecting arginine and lysine amino acids. *Dyes Pigm.* **2020**, *175*, 108131.
9. Zhu, Z; Wang, Y.; Ding, H.; Fan, C.; Tu, Y.; Liu, G; Pu, S. A novel full symmetric diarylethene-based ratiometric fluorescent sensor for lysine and the application for a logic circuit. *Luminescence* **2021**, *36*, 691-697.
10. Yang, L.; Xie, Y.; Chen, Q.; Zhang, J.; Li, L.; Sun, H. Colorimetric and fluorescent dual-signal chemosensor for lysine and arginine and its application to detect amines in solid-phase peptide synthesis. *ACS Appl. Bio Mater.* **2021**, *4*, 6558-6564.
11. Gong, Y.; Du, C.; Wang, X.; Guo, H.; Yang, F. First stable (Z)-configuration of cyanostilbene derivative: An effective "turn-on" fluorescent sensor for lysine in aqueous media. *Microchem. J.* **2021**, *162*, 105866.
12. Wang, T.; Pang, Q.; Tong, Z.; Xiang, H.; Xiao, N. A hydrazone-based spectroscopic off-on probe for sensing of basic arginine and lysine. *Spectrochim. Acta, Part A* **2021**, *258*, 119824.
